# Risk Factors for Acquisition of Fluoroquinolone or Aminoglycoside Resistance in Addition to Carbapenem Resistance in *Pseudomonas Aeruginosa*

**DOI:** 10.2174/1874285801711010092

**Published:** 2017-05-31

**Authors:** Kosuke Kosai, Norihito Kaku, Naoki Uno, Tomomi Saijo, Yoshitomo Morinaga, Yoshifumi Imamura, Hiroo Hasegawa, Taiga Miyazaki, Koichi Izumikawa, Hiroshi Mukae, Katsunori Yanagihara

**Affiliations:** 1 Department of Laboratory Medicine, Nagasaki University Hospital, Nagasaki, Japan; 2 Department of Laboratory Medicine, Nagasaki University Graduate School of Biomedical Sciences, Nagasaki, Japan; 3 Department of Respiratory Medicine, Nagasaki University Graduate School of Biomedical Sciences, Nagasaki, Japan; 4 Department of Infectious Diseases, Nagasaki University Graduate School of Biomedical Sciences, Nagasaki, Japan

**Keywords:** Metallo-β-lactamase, Drug resistance, Infection control, Antibiotic therapy, AG resistance

## Abstract

**Background:**

Carbapenems, fluoroquinolones (FQs), and aminoglycosides (AGs) are key drugs for treating *Pseudomonas aeruginosa* infections, and accumulation of drug resistances make antibiotic therapy difficult.

**Methods:**

We evaluated 169 patients with imipenem (IPM)-resistant *P. aeruginosa* and compared patient background and microbiological characteristics between groups with or without FQ resistance. Similar analyses were performed for AG.

**Results:**

Of the 169 IPM-resistant strains, 39.1% showed resistance to FQs and 7.1% to AGs. The frequency of exposure to FQs within 90 days previously was higher in the group with FQ resistance (45.5%) than in the group without FQ resistance (13.6%). Similarly, 33.3% of patients in the group with AG resistance had been previously administered AGs, higher than the 7.6% of patients without AG resistance. Frequencies of metallo-β-lactamase (MBL) production were higher in the group with FQ or AG resistance (16.7% or 33.3%) than in the group without FQ or AG resistance (2.9% or 6.4%). Multivariate analyses showed exposures to FQs or AGs were related to the respective resistances. MBL production was a common factor for resistance to FQs or AGs, in addition to IPM-resistant *P. aeruginosa*.

**Conclusion:**

As well as promoting appropriate use of antibiotics, MBL production should be detected as a target of intervention for infection control.

## 1 INTRODUCTION

*Pseudomonas aeruginosa* is an important pathogen for nosocomial infections. *P. aeruginosa* displays not only intrinsic resistance, but also the ability to acquire resistance during antibiotic therapy [1]. Furthermore, the acquisition of drug-resistant pathogens by an individual patient involves numerous factors, such as microbial selection by antibiotic pressures and in-hospital transmission from other patients or medical environments.

Carbapenems, fluoroquinolones (FQs), and aminoglycosides (AGs) are key drugs for treating *P. aeruginosa* infections, and accumulation of drug resistances make antibiotic therapy difficult. The Japan Nosocomial Infections Surveillance (JANIS), a program of the Ministry of Health Labour and Welfare, reported in 2013 that 78.3% and 84.0% of *P. aeruginosa* strains were susceptible to imipenem (IPM) and meropenem, 84.0% and 94.8% to gentamicin and amikacin, and 78.6% to levofloxacin, respectively [2] These susceptibility rates have been gradually improving in recent years in Japan, but drug-resistant *P. aeruginosa* remains an important issue from the perspective of patient outcomes and infection control [3, 6].

A previous study reported that the mortality rate for ventilator-associated pneumonia due to *P. aerugii.e.nosa* depended on the adequacy of initial empiric therapy in terms of susceptibility, regardless of the use of monotherapy or combination therapy. Furthermore, the use of a β-lactam plus FQ or AG was the major therapeutic option if combination therapy was empirically selected [4]. In addition, empiric combination therapy may improve patient outcomes, by increasing the likelihood of adequate coverage [5].

Whether *P. aeruginosa* displays resistance to FQs or AGs in addition to β-lactams is therefore considered critical as a preliminary step to multidrug-resistance. However, few studies have evaluated this issue in detail. The objective of this study was to investigate risk factors for the acquisition of resistance to FQs or AGs in imipenem-resistant *P. aeruginosa*.

## 2 METHODS

### Study Design and Clinical Data Collection

We investigated 169 patients from whom IPM-resistant strains of *P. aeruginosa* were isolated at Nagasaki University Hospital between January 2010 and December 2012. If IPM-resistant *P. aeruginosa* was repeatedly identified from a single patient during the study period, de-duplication was performed. First, strains were selected by the priority order according to the pattern of resistance, as follows: 1) presence of resistance to both FQs and AGs; 2) presence of resistance to either FQs or AGs; or 3) presence of resistance to neither FQs nor AGs. The first identified strain among those with the same pattern of resistance was then included in this study. This process was performed regardless of specimen type.

Patients were divided into two groups according to the presence or absence of resistance to FQs (ciprofloxacin and/or levofloxacin). We compared patient backgrounds and microbiological characteristics between groups. Furthermore, we analysed risk factors for the acquisition of FQ resistance in IPM-resistant *P. aeruginosa*. Similar analyses were then carried out for AGs (gentamicin and/or amikacin).

We evaluated the presence of exposure to intravenously administered antibiotics within 90 days prior to the identification of IPM-resistant *P. aeruginosa*. Antipseudomonal penicillins (APPs) included piperacillin and piperacillin/tazobactam. Cefmetazole and flomoxef were included as second-generation cephalosporins in this study.

This study was approved by the institutional review board of Nagasaki University Hospital. Informed consent was not required because the study was retrospective and the data were obtained within the context of normal daily practice.

### Antimicrobial Susceptibility Testing and Detection of Metallo-β-Lactamase

Susceptible, intermediate, and resistant strains were decided according to Clinical and Laboratory Standard Institute (CLSI) M100-S21. Bacterial identification and minimum inhibitory concentration (MIC) measurements were performed using a BD Phoenix automated microbiology system (BD Diagnostics, Sparks, MD). If the MIC for ceftazidime (CAZ) against *P. aeruginosa* was ≥ 32 µg/mL and that of IPM was ≥ 8 µg/mL, metallo-β-lactamase (MBL) was examined using an MIC plate with CAZ containing 400 µg/mL of sodium mercaptoacetate (SMA). Ranges for MIC measurements were 16-128 µg/mL for CAZ and 8-32 µg/mL for CAZ with SMA in the plate. Bacterial isolates were considered as MBL producers if the MIC of CAZ was reduced by three or more doubling dilutions in the presence of SMA.

### Statistical Analysis

The difference in age between groups was analysed using Mann-Whitney U test, because age was not normally distributed. Fisher’s exact test was used to compare categorical data between groups. We conducted uni- and multivariate analyses using a logistic regression model. Variables with values of P < 0.2 in univariate analysis were selected and adjusted by forward stepwise selection in multivariate analysis to identify risk factors for the acquisition of resistance to FQs or AGs. Data were analysed using SPSS for Windows version 16.0J (SPSS, Chicago, IL) and P values of 0.05 were considered statistically significant.

## RESULTS

### Patient Characteristics

Details of the 169 patients enrolled in the study are presented in Table **1**. Of the 169 IPM-resistant strains, 66 strains (39.1%) showed resistance to FQs. Frequencies of exposure to FQs and AGs within 90 days previously were significantly higher for the group with FQ resistance (45.5% and 16.7%, respectively) than in the group without FQ resistance (13.6%, P < 0.001 and 4.9%, P = 0.015, respectively). Conversely, 4.5% of patients in the group with FQ resistance had been administered a second-generation cephalosporin, lower than the 14.6% of patients without FQ resistance (P = 0.043).

Twelve strains (7.1%) showed AG resistance. The frequency of exposure to AGs within 90 days previously was higher in the AG-resistant group (33.3%) than in the group without AG resistance (7.6%, P = 0.017).

No significant differences between the groups with or without FQ resistance were seen in age, sex, identification at ≥30 days after hospitalization, comorbidities, use of immunosuppressive drugs (anticancer drugs, steroid or other immunosuppressive agents) and use of medical devices. Similar results were seen in comparisons between groups with or without AG resistance.

### Antimicrobial Susceptibility and MBL Production

Fig. (**1**) shows the resistance rates of strains stratified by the presence of FQ or AG resistance. The rates of resistance to β-lactams and gentamicin were significantly higher in strains with FQ resistance than in those without FQ resistance. Strains with AG resistance had higher rates of resistance to ceftazidime, cefepime and ciprofloxacin, compared with strains without AG resistance. MBL-producing strains were seen more frequently in strains with FQ or AG resistance (16.7% or 33.3%, respectively) than in those without FQ or AG resistance (2.9%, P = 0.003 or 6.4%, P = 0.010, respectively).

### Risk Factors for Acquisition of FQ or AG Resistance

Univariate and multivariate logistic regression analyses of risk factors for acquisition of FQ or AG resistance are shown in Table **2**. Exposure to FQs or AGs showed clear relationships to resistances to those respective drug classes [odds ratio (OR), 5.73; 95% confidence interval (CI), 2.67–12.30 or OR, 9.00; 95% CI, 1.92–42.19, respectively]. In contrast, previous use of APPs reduced the risk of acquiring AG resistance. MBL was a common factor associated with resistance to FQs or AGs in IPM-resistant *P. aeruginosa* (OR, 7.90; 95% CI, 2.01–31.04 or OR, 8.49; 95% CI, 1.87–38.52, respectively).

## DISCUSSION

Our results indicated antimicrobial exposures as risk factors for the acquisition of FQ or AG resistance in IPM-resistant *P. aeruginosa*, supporting previous report [1]. Furthermore, MBL represented an independent factor for the accumulation of FQ or AG resistance in addition to IPM resistance, although production of MBL was not a direct contributor to FQ or AG resistance. Several mechanisms underlying drug resistance have been reported, such as efflux pump and co-production of both IMP-type MBL and aminoglycoside acetyl-transferases [7]. Accumulation of drug resistance would involve these mechanisms, along with propagation of plasmids for resistant genes and antibiotic selection pressure. The reason why previous use of APPs correlated negatively with AG resistance remains unclear.

Several host factors, medical devices and medical environments have been reported as risk factors for drug-resistant *P. aeruginosa* [8]. However, no differences in patient backgrounds other than previous use of antibiotics were evident between the groups with and without FQ or AG resistance in this study. This was attributed to the patients included in this study having already acquired IPM-resistant *P. aeruginosa*, with the divided groups representing relatively similar populations with regard to host and medical backgrounds.

Some limitations must be considered when interpreting the present results. First, prevalence of drug-resistant *P. aeruginosa* and rates of strains producing MBL would vary widely by geographic region. Our results might therefore not be applicable to other institutions. Second, because of our de-duplication processes, rates of FQ or AG resistance among strains with IPM-resistance would have seemed higher than they actually were. Third, since other mechanisms of drug-resistance were not evaluated, particularly with regard to FQs and AGs, the direct relationship between MBL production and FQ or AG resistance remains unclear. Additionally, not all MBL producers could be detected, because ranges of MIC measurement were limited in CAZ and CAZ/SMA for MBL detection and MBL genes were not evaluated. As our previous study reported, use of real-time polymerase chain reactions would be useful for detecting *P. aeruginosa* and MBL gene [9].

This study identified antimicrobial exposure and production of MBL as independent risk factors for FQ or AG resistance in IPM-resistant *P. aeruginosa* in our hospital setting. As well as promoting appropriate use of antibiotics, MBL production should be detected as a target of intervention for infection control, because this function is spreading between bacterial species and represents a risk factor for the accumulation of drug resistance. Quick and accurate detection of MBL genes as with phenotype should be performed as needed.

## Figures and Tables

**Fig. (1):**
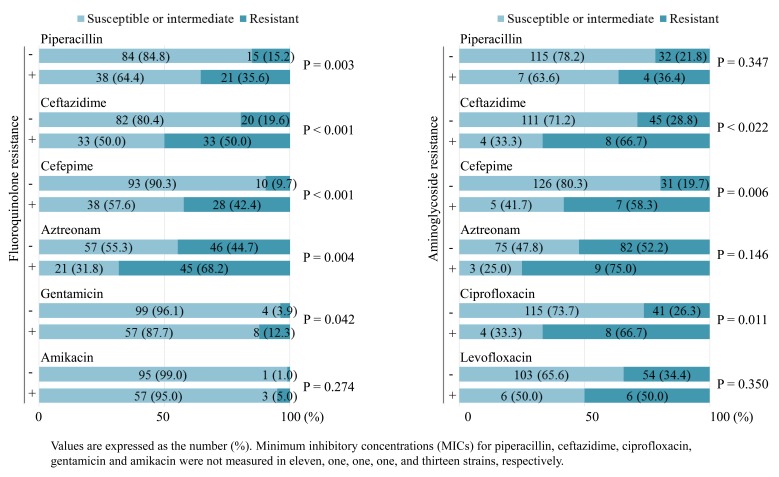
The resistance rates of strains stratified by the presence of fluoroquinoloneor aminoglycosideresistance in imipenem-resistant *Pseudomonas aeruginosa*.

**Table 1:** Baseline characteristics of patients with imipenem-resistant *pseudomonas aeruginosa* stratified by presence of FQ or AG resistance.

		Resistance to FQs			Resistance to AGs		
	All patients (n = 169)	Yes (n = 66)	No (n = 103)	P	Yes (n = 12)	No (n= 157)	P
Age (years)	66.3 ± 15.1	64.5 ± 15.2	67.5 ± 15.0	0.228	62.7 ± 17.8	66.6 ± 14.9	0.514
Sex (male / female)	115/54 (68.0)	43/23 (65.2)	72 /31 (69.9)	0.612	9/3 (75.0)	106/51 (67.5)	0.754
Identification at ≥30 days after hospitalization	82 (48.5)	35 (53.0)	47 (45.6)	0.430	7 (58.3)	75 (47.8)	0.557
Comorbidities and conditions							
Malignancy	73 (43.2)	25 (37.9)	48 (46.6)	0.271	6 (50.0)	67 (42.7)	0.764
Diabetes	37 (21.9)	17 (25.8)	20 (19.4)	0.346	3 (25.0)	34 (21.7)	0.726
Chronic dialysis	13 (7.7)	5 (7.6)	8 (7.8)	1.000	0 (0.0)	13 (8.3)	0.602
Transplantation	32 (18.9)	16 (24.2)	16 (15.5)	0.166	2 (16.7)	30 (19.1)	1.000
Anticancer drugs	21 (12.4)	10 (15.2)	11 (10.7)	0.475	3 (25.0)	18 (11.5)	0.174
Corticosteroids (≥5 mg/day) or other immunosuppressive agents	51 (30.2)	25 (37.9)	26 (25.2)	0.088	2 (16.7)	49 (31.2)	0.514
Surgery	98 (58.0)	34 (51.5)	64 (62.1)	0.202	6 (50.0)	92 (58.6)	0.562
Medical devices							
Central venous catheter	81 (47.9)	29 (43.9)	52 (50.5)	0.433	4 (33.3)	77 (49.0)	0.375
Tracheal tube	66 (39.1)	26 (39.4)	40 (38.8)	1.000	3 (25.0)	63 (40.1)	0.370
Ventilator	41 (24.3)	19 (28.8)	22 (21.4)	0.277	1 (8.3)	40 (25.5)	0.297
Urinary catheter	104 (61.5)	42 (63.6)	62 (60.2)	0.746	5 (41.7)	99 (63.1)	0.217
Feeding tube	87 (51.5)	33 (50.0)	54 (52.4)	0.875	6 (50.0)	81 (51.6)	1.000
Exposure to antibiotics within 90 days							
Antipseudomonal penicillins	73 (43.2)	30 (45.5)	43 (41.7)	0.637	2 (16.7)	71 (45.2)	0.071
Other penicillins	47 (27.8)	20 (30.3)	27 (26.2)	0.600	4 (33.3)	43 (27.4)	0.740
First-generation cephalosporins	52 (30.8)	16 (24.2)	36 (35.0)	0.173	2 (16.7)	50 (31.8)	0.347
Second-generation cephalosporins	18 (10.7)	3 (4.5)	15 (14.6)	0.043	1 (8.3)	17 (10.8)	1.000
Third-generation cephalosporins	45 (26.6)	20 (30.3)	25 (24.3)	0.476	2 (16.7)	43 (27.4)	0.519
Fourth-generation cephalosporins	14 (8.3)	7 (10.6)	7 (6.8)	0.403	2 (16.7)	12 (7.6)	0.260
Carbapenem	119 (70.4)	45 (68.2)	74 (71.8)	0.610	8 (66.7)	111 (70.7)	0.750
Monobactam	2 (1.2)	2 (3.0)	0 (0.0)	0.151	0 (0.0)	2 (1.3)	1.000
FQs	44 (26.0)	30 (45.5)	14 (13.6)	<0.001	4 (33.3)	40 (25.5)	0.513
AGs	16 (9.5)	11 (16.7)	5 (4.9)	0.015	4 (33.3)	12 (7.6)	0.017

Values are expressed as mean ± standard deviation or the number (%).

FQs, fluoroquinolones; AGs, aminoglycosides

**Table 2:** Univariate and multivariate analyses of risk factors for acquisition of fluoroquinolone or aminoglycoside resistance in addition to imipenem resistance in *Pseudomonas aeruginosa*.

	Univariate analysis			Multivariate analysis		
	OR	95%CI	P	OR	95%CI	P
Resistance to FQs						
Transplantation	1.74	0.80-3.78	0.161			
Use of corticosteroids (≥5 mg/day) or other immunosuppressive agents	1.81	0.93-3.52	0.082			
Surgery	0.65	0.35-1.21	0.173			
Exposure to first-generation cephalosporins	0.60	0.30-1.19	0.143			
Exposure to second-generation cephalosporins	0.28	0.08-1.01	0.051			
Exposure to FQs	5.30	2.52-11.14	<0.001	5.73	2.67-12.30	<0.001
Exposure to AGs	3.92	1.30-11.87	0.016			
MBL production	6.67	1.78-24.91	0.005	7.90	2.01-31.04	0.003
Resistance to AGs						
Use of anticancer drugs	2.57	0.64-10.40	0.184			
Use of urinary catheter	0.42	0.13-1.38	0.152			
Exposure to antipseudomonal penicillins	0.24	0.05-1.14	0.073	0.16	0.03-0.89	0.036
Exposure to AGs	6.04	1.59-23.00	0.008	9.00	1.92-42.19	0.005
MBL production	7.35	1.89-28.65	0.004	8.49	1.87-38.52	0.006

OR, odds ratio; CI, confidence interval; FQs, fluoroquinolones; AGs, aminoglycosides; MBL, metallo-β-lactamase

## ADDITIONAL INFORMATION

This work was presented as an ePoster at the 25^th^ European Congress of Clinical Microbiology and Infectious Diseases (ECCMID).
